# Clinical characteristics and histopathology of COVID-19 related deaths in South African adults

**DOI:** 10.1371/journal.pone.0262179

**Published:** 2022-01-20

**Authors:** Marta C. Nunes, Martin J. Hale, Sana Mahtab, Fikile C. Mabena, Noluthando Dludlu, Vicky L. Baillie, Bukiwe N. Thwala, Toyah Els, Jeanine du Plessis, Marius Laubscher, Shakeel Mckenzie, Sihle Mtshali, Colin Menezes, Natali Serafin, Sarah van Blydenstein, Merika Tsitsi, Brian Dulisse, Shabir A. Madhi

**Affiliations:** 1 South African Medical Research Council, Vaccines and Infectious Diseases Analytics Research Unit, School of Pathology, Faculty of Health Sciences, University of the Witwatersrand, Johannesburg, South Africa; 2 Department of Science and Technology/National Research Foundation, South African Research Chair Initiative in Vaccine Preventable Diseases, Faculty of Health Sciences, University of the Witwatersrand, Johannesburg, South Africa; 3 Division of Anatomical Pathology, School of Pathology, Faculty of Health Sciences, University of the Witwatersrand, Johannesburg, South Africa; 4 Department of Paediatrics and Child Health, Chris Hani Baragwanath Academic Hospital, Faculty of Health Sciences, University of the Witwatersrand, Johannesburg, South Africa; 5 Department of Internal Medicine, Chris Hani Baragwanath Academic Hospital, Faculty of Health Sciences, University of the Witwatersrand, Johannesburg, South Africa; 6 BDII Analytics, Atlanta, GA, United States of America; 7 African Leadership in Vaccinology Expertise, Faculty of Health Sciences, University of the Witwatersrand, Johannesburg, South Africa; University of Hail, SAUDI ARABIA

## Abstract

Comparisons of histopathological features and microbiological findings between decedents with respiratory symptoms due to SARS-CoV-2 infection or other causes, in settings with high prevalence of HIV and *Mycobacterium tuberculosis* (MTB) infections have not been reported. Deaths associated with a positive ante-mortem SARS-CoV-2 PCR test and/or respiratory disease symptoms at Chris Hani Baragwanath Academic Hospital in Soweto, South Africa from 15th April to 2nd November 2020, during the first wave of the South African COVID-19 epidemic, were investigated. Deceased adult patients had post-mortem minimally-invasive tissue sampling (MITS) performed to investigate for SARS-CoV-2 infection and molecular detection of putative pathogens on blood and lung samples, and histopathology examination of lung, liver and heart tissue. During the study period MITS were done in patients displaying symptoms of respiratory disease including 75 COVID-19-related deaths (COVID+) and 42 non-COVID-19-related deaths (COVID-). The prevalence of HIV-infection was lower in COVID+ (27%) than in the COVID- (64%), MTB detection was also less common among COVID+ (3% vs 13%). Lung histopathology findings showed differences between COVID+ and COVID- in the severity of the morphological appearance of Type-II pneumocytes, alveolar injury and repair initiated by SARS-CoV-2 infection. In the liver necrotising granulomatous inflammation was more common among COVID+. No differences were found in heart analyses. The prevalence of bacterial co-infections was higher in COVID+. Most indicators of respiratory distress syndrome were undifferentiated between COVID+ and COVID- except for Type-II pneumocytes. HIV or MTB infection does not appear in these data to have a meaningful correspondence with COVID-related deaths.

## Introduction

The coronavirus disease 2019 (COVID-19) pandemic, caused by Severe Acute Respiratory Syndrome Coronavirus 2 (SARS CoV-2), has caused more than 5.3 million deaths as of November 2021 [1]. These deaths are often associated with respiratory illness, e.g. pneumonia and acute respiratory distress syndrome (ARDS). Post-mortem studies have documented a link between aspects of respiratory distress syndrome, such as diffuse alveolar damage and infection with SARS-CoV-2 [2–4]. These studies have, for the most part, focused on geographic areas other than Africa, and thus feature populations with generally lower background rates of various diseases, such as underlying HIV infection and tuberculosis [5, 6]; with only single case reports or studies with small number of COVID-19 patients being available from the African continent [7–10]. Furthermore, possible differences in genetics, social behaviours and exposures to other infectious agents in the population may affect the pathology due to SARS-CoV-2 infection.

Pathologic studies of decedents may provide important information related to disease presentation, therapeutic decision making, and inform trials of strategies designed to prevent severe disease [11]. In the early part of the pandemic, case reports on individual or small numbers of decedents were published [12–15]. Subsequently, reviews of collections of such smaller studies began to appear [16, 17]. Recently, studies of larger numbers of decedents and meta-analyses of smaller studies have been published [18, 19]. Because ARDS is common in severely ill patients with SARS-CoV-2 infections, the large majority of the literature has focused on the analysis of lung tissue samples [20, 21], with only a few study reporting on multiple tissue findings [19, 22, 23]. This study documents post-mortem lung, liver and heart histopathology, and blood and lung culture and molecular assay findings among adult decedents in Soweto, South Africa diagnosed with respiratory symptoms, and compares these findings based on SARS-CoV-2 infection status diagnosed either ante-mortem or post-mortem.

## Methods

Chris Hani Baragwanath Academic Hospital (CHBAH) is one of two public hospitals serving the population of Soweto, Gauteng, South Africa, providing tertiary-level hospital care to a population that generally (>90%) depends on the public health service. Details of the population of Soweto and the standard of care practices at the CHBAH have been published previously [24]. The first SARS-CoV-2 case in South Africa was reported on 5^th^ March 2020 and, subsequently, the first known hospitalized case of COVID-19 at CHBAH was diagnosed on 6^th^ April 2020. The number of COVID-19 cases admitted at CHBAH was low until the end of May 2020, after which the numbers rapidly increased with infections peaking from mid-June to early August (week 26 to 32), the number of COVID-19 cases was again low during October and November 2020 [25]. This study was conducted from 15^th^ April to 2^nd^ November 2020.

For this study, the families of the patients dying in-hospital with a positive ante-mortem SARS-CoV-2 polymerase chain reaction (PCR) test and/or respiratory disease symptoms during the hospital stay were contacted after the patient’s death to obtain permission for inclusion in the study. Decedents were considered to have displayed respiratory symptoms if they had been diagnosed with bronchopneumonia, tuberculosis, bronchitis, lower respiratory tract infection, asthma/acute exacerbation of asthma, chronic obstructive pulmonary disease, or lung disease, or if a clinical finding of respiratory distress, tachypnoea, stridor, chest wall retraction, wheeze, bronchial breathing, or two or more of cough, shortness of breath, or difficulty breathing. For each decedent for whom consent was obtained, medical record information was collected on demographic characteristics, duration of hospital stay, HIV infection status and other comorbidities.

Post-mortem minimally-invasive tissue sampling (MITS) was used in place of full autopsies. MITS has been validated against complete diagnostic autopsy and has shown high concordance for attributing cause of death, particularly for infectious diseases [26]. MITS sampling was undertaken within 72 hours of death and included a mid-turbinate nasal swab for SARS-CoV-2 PCR testing, blood collection, as well as core biopsy tissue sampling of the lung, liver, and heart with the aim of at least 6 cores obtaining target tissues at each site, with 2 cores being considered adequate. Tissue samples were sent for histopathological examination, and lung tissue samples were also sent for bacterial culture and molecular detection of respiratory pathogens (including SARS-CoV-2). Blood was collected into EDTA tubes following decontamination of the skin surface through cardiac puncture or by supraclavicular approach into the left subclavian vessel. Blood was then inoculated into Bact/ALERT PF Plus bottle and evaluated using the BacT/Alert microbial system (BioMerieux, Marcy l’Etoile, France) at the National Health Laboratory Services at CHBAH. All positive cultures from the various sample types were Gram stained and further identified using standard manual methods. Gram staining, culture identification and antibiotic susceptibility testing were done according to clinical laboratory standard institute guidelines following culture.

Following a biopsy, tissues for histopathology were immediately placed in 10% neutral buffered formalin and fixed for a minimum period of 6 hours, but no longer than 24 hours. After fixation, the biopsies were dehydrated in graded ethanol concentrations, cleared in xylene, and infiltrated with wax, using the Tissue-Tek Vacuum Infiltration Processor manufactured by Sakura. Tissues were then embedded in wax blocks from which 4 micron sections were cut and stained with haematoxylin and eosin (H&E). Protocols for the tissue processing and H&E staining were based on widely established protocols and methodology [27]. A histopathologist, blinded to clinical history and laboratory findings, examined the stained tissue sections using an Olympus BX41 light microscope. Owing to the limited quantities of tissue obtained by the MITS procedure, the histopathological assessment of the pathological changes was confined to the interpretation of the H&E stain so tissue could be preserved for future immunohistochemical and molecular investigation.

Nucleic acid amplification test using the Emergency use authorization assay developed by the USA Centers for Diseases Control and Prevention (CDC) was used to detect SARS-CoV-2 in respiratory and blood samples. Results were classified as positive for SARS-CoV-2 when both the N1 and N2 targets of the nucleocapsid gene were detected by PCR with cycle threshold (Ct) values were <40.

The presence and abundance of specific viruses, bacteria and non-bacterial agents in lung tissue were also evaluated using PCR based methods. Real-time PCR was performed using the Open Array QuantStudio 12k Flex Real-Time PCR System with TaqMan Array Cards (TAC) according to manufacturer’s instructions (Thermofisher Scientific, Waltham, MA, USA) and the pathogens investigated are listed in S1 Table.

GeneXpert testing for detection of *Mycobacterium tuberculosis* (MTB) was performed as previously described [28]. Briefly, sample reagent was added in a 3:1 ratio to 0.5mL of lysed lung biopsy samples. Incubated for 15 minutes with periodic agitation before being transferred to the Xpert MTB/RIF Ultra test cartridge. Cartridges were loaded into the GeneXpert device and the results were generated automatically.

This is a descriptive report on a convenience sampling and no formal statistical significance was evaluated. Among SARS-CoV-2 decedents stratifications by HIV-infection status and duration of hospital stay before death (≤3 days vs >3 days) are presented.

### Ethics considerations

The study was approved by the Human Research Ethics Committee at the University of the Witwatersrand (HREC approval number: M200313). Informed written consent was obtained from relatives of the deceased.

## Results

From 15^th^ April to 2^nd^ November 2020, there were 600 deaths with a positive SARS-CoV-2 polymerase chain reaction (PCR) test ante-mortem at Chris Hani Baragwanath Academic Hospital (CHBAH); of these, post-mortem minimally-invasive tissue sampling (MITS) were performed on 68. Additionally, MITS were done in 11 decedents who had a negative ante-mortem test but a positive post-mortem test and 2 SARS-CoV-2 positive decedents who were just tested post-mortem, for a total of 81 SARS-CoV-2 infected decedents. Of these 81 decedents, six tested SARS-CoV-2 ante-mortem but did not manifest any respiratory symptoms. Because the focus of this analysis is the comparison between SARS-CoV-2 infected and–uninfected decedents with respiratory symptoms, these 6 were not included in the analysis. The 75 decedents displaying symptoms of respiratory disease along with one or more positive SARS-CoV-2 tests are referred to as the COVID-positive (COVID+) group. Furthermore, 42 decedents who tested negative for SARS-CoV-2 on ante- or post-mortem sampling were also included; all these displayed symptoms of respiratory disease and are for convenience referred to as the COVID-negative (COVID-) group.

The COVID+ decedents were slightly older than the COVID- group (60 vs 51 years), and more likely to be female (46% vs 23%). Hypertension (54% vs 40%) and diabetes (37% vs 16%) were the two comorbidities more common among COVID+ decedents compared to those in the COVID- group. There was a lower prevalence of HIV-infection in the COVID+ group (27%) than in the COVID- group (64%) (Table 1). Median hospital duration before death was 5 days in the COVID+ group (median 4 days for the decedents with negative ante-mortem test and positive post-mortem test) and 4 days in the COVID- group.

**Table 1:** Baseline characteristics of decedents presenting with respiratory illness stratified by SARS-CoV-2 infection status either on ante- or post-mortem testing.

	COVID+	COVID‒
	n = 75	n = 42
Median age in yeas (IQR)	60 (49, 68)	51 (42, 65)
Gender		
Male	29 (39)	19 (45)
Female	46 (61)	23 (55)
HIV-infected	20 (27)	27 (64)
Median number of days of hospitalization (IQR)	5 (1, 12)	4 (1, 7)
Median hours between death and MITS (IQR)	30 (27, 49)	31 (27, 38)
Comorbidities		
Asthma	4 (5)	1 (2)
Arthritis	1 (1)	1 (2)
Chronic obstructive pulmonary disease	6 (8)	6 (14)
Other chronic lung disease	2 (3)	0
Cardiovascular disease	3 (4)	1 (2)
Hypertension	41 (54)	17 (40)
Cerebrovascular accident/stroke	6 (8)	2 (5)
Diabetes	28 (37)	7 (16)
Cancer	2 (3)	2 (5)
Epilepsy	3 (4)	0
Renal failure	18 (24)	10 (24)
Preexisting	9 (12)	6 (14)
Current	9 (12)	4 (10)
Other organ disease	2 (3)	1 (2)
SARS-CoV-2 PCR result		
Ante-mortem	63 (84)	
Post-mortem—nasal swab	54 (72)	
Post-mortem—lung	59 (77)	
Post-mortem—blood	43 (57)	

Results are n (%) unless stated otherwise.

COVID+: positive for SARS-CoV-2 on PCR testing; COVID-: SARS-CoV-2 not identified on PCR testing.

IQR: interquartile range.

Table 2 gives details of the histopathological diagnostic features in COVID+ and COVID- decedents. Relatively few of the histopathological features differed between the two groups. The COVID+ group, however, showed greater severity in each phase of the lung alveolar injury/repair cycle, irrespective as to whether the changes were assessed using the established classification of acute respiratory distress syndrome into the exudative, regenerative and organization phases, or a more recent classification utilizing a cellular approach (epithelial, vascular, fibrotic) [29–31].

**Table 2:** Histopathological diagnostic characteristics in decedents hospitalized with respiratory illness with (COVID+) and without (COVID‒) SARS-CoV-2 infection.

	COVID+	COVID-
**Lung**	**n = 72**	**n = 42**
Bronchopneumonia (bacterial pneumonia or bronchopneumonia without infectious agent)	12 (17)	6 (14)
Acute respiratory distress syndrome	57 (79)	22 (52)
Viral pneumonia (viral or interstitial pneumonitis)	66 (92)	34 (81)
Fungal pneumonia	0 (0)	1 (2)
Aspiration pneumonia	2 (3)	1 (2)
Granulomas, necrotizing	3 (4)	6 (14)
Granulomas, non-necrotizing	1 (1)	0 (0)
Corrin & Nicholson classification [30]		
Exudative phase	45 (63)	14 (33)
Regenerative phase	51 (71)	20 (48)
Repair phase	49 (68)	18 (43)
Polak et al classification [29]		
Epithelial phase	54 (75)	20 (48)
Vascular phase	23 (32)	9 (21)
Fibrotic phase	49 (68)	18 (43)
**Liver**	**n = 74**	**n = 41**
Steatosis	10 (14)	5 (12)
Steatohepatitis	4 (5)	0 (0)
Sinusoidal leukocytosis	25 (34)	14 (34)
Sepsis	4 (5)	3 (7)
Cholestasis	8 (11)	2 (5)
Viral hepatitis	1 (1)	0 (0)
Active hepatitis	12 (16)	6 (15)
Chronic hepatitis	1 (1)	0 (0)
Granulomatous hepatitis	1 (1)	5 (12)
Fibrosis	1 (1)	1 (2)
Cirrhosis	1 (1)	0 (0)
Fibrin-platelet thrombi	7 (9)	1 (2)
**Heart**	**n = 68**	**n = 37**
Neutrophilc myocarditis	1 (1)	2 (5)
Lymphocytic myocarditis	6 (9)	5 (14)
Vasculitis	2 (3)	0 (0)
Fibrin platelet thromboembolic disease	2 (3)	1 (3)
Myocardial fibre hypertrophy/cardiomegaly	37 (54)	18 (49)

Results are n (%).

The numbers provided will not match those provided in the tables detailing organ specific findings since the later record a finding but not necessarily reflecting a diagnosis.

Table 3 and S1 Fig presents more detailed lung histological findings. The morphological changes in the lung that were 1.5 to over 10 times more common in the COVID+ than the COVID- group included: organizing pneumonia (31% vs 10%), hyaline membrane formation (64% vs 29%), overall pneumocyte proliferation (85% vs 62%), and specifically cytomegaly (76% vs 45%), nucleomegaly (75% vs 45%), multinucleation (63% vs 38%) and syncytia formation (33% vs 2%). Stratifying the COVID+ group by HIV infection status did not reveal major differences between HIV status, with only intra-alveolar fibrin being more common in HIV-infected (60%) compared with HIV-uninfected (38%) decedents (S2 Table). Stratification of the COVID+ group by duration of hospital stay, showed that neutrophilic infiltrate (35% vs 16%) and intra-alveolar hemosiderosis (28% vs 0%) were more common in those hospitalised for >3 days compared with hospitalizations ≤3 days (S3 Table).

**Table 3:** Histopathology lung features in decedent66ts hospitalized with respiratory illness with (COVID+) and without (COVID-) SARS-CoV-2 infection.

	COVID+	COVID-
	n = 75	n = 42
Necrotizing granulomata	5 (7)	7 (17)
Non-necrotizing granulomas	0 (0)	1 (2)
Fungi	0 (0)	1 (2)
Aspirated material	1 (1)	1 (2)
Neutrophilic infiltrate	20 (28)	13 (31)
Intra-alveolar hemosiderosis	12 (17)	10 (24)
Congestion of alveolar septa	66 (92)	37 (88)
Alveolar septal oedema	60 (83)	28 (67)
Interstitial inflammation	67 (93)	38 (90)
Intravascular fibrin/microthrombi	25 (35)	12 (29)
Megakaryocytes	43 (60)	23 (55)
Intra-alveolar hemorrhage	15 (21)	5 (12)
Intra-alveolar oedema	16 (22)	5 (12)
Intra-alveolar fibrin	33 (46)	16 (38)
Hyaline membranes	46 (64)	12 (29)
Alveolar collapse	48 (67)	23 (55)
Type II pneumocyte overall	61 (85)	26 (62)
Type II pneumocyte morphology-Cytomegaly	55 (76)	19 (45)
Type II pneumocyte morphology-Nucleomegaly	53 (74)	19 (45)
Type II pneumocyte morphology-Multinucleation	45 (63)	16 (38)
Type II pneumocyte proliferation-Syncytia formation	24 (33)	1 (2)
Increased alveolar macrophages	63 (88)	37 (88)
Alveolar septal necrosis	61 (85)	35 (83)
Intra-alveolar or Septal collagen or honeycombing	60 (83)	39 (93)
Intra-alveolar organization/organizing pneumonia	22 (31)	4 (10)
Septal collagen deposition	57 (79)	37 (88)
Honeycombing/microcystic fibrosis	7 (10)	3 (7)

Results are n (%).

Table 4 presents results on post-mortem lung TaqMan Array Cards (TAC) and *Mycobacterium tuberculosis* (MTB) GeneXpert testing. Overall, more bacteria were detected among the COVID+ group compared to the COVID- group, especially for *Acinetobacter baumannii* (12% vs 5%), *Escherichia coli* (11% vs 0%) and *Klebsiella pneumoniae* (12% vs 2%). *Pneumocystis jirovecii* was however only detected in COVID- decedents (10%, all of whom were also HIV-infected). Detection of MTB was more common among the COVID- (13%) than the COVID+ group (3%); all seven patients positive for GeneXpert showed necrotizing granulomatous inflammation on histopathological examination. Stratification of the COVID+ group by HIV-infection status showed a similar frequency of detection for most pathogens with only Cytomegalovirus (15% vs 4%) and MTB (both samples positive on GeneXpert test were obtained from HIV-infected decedents, 11% vs 0%) being more frequently detected in HIV-infected decedents (S4 Table).

**Table 4:** Organisms identified on post-mortem lung molecular assay detection in decedents hospitalized with respiratory illness with (COVID+) and without (COVID‒) SARS-CoV-2 infection.

	COVID+	COVID‒
	n = 75	n = 42
Bacteria		
*Acinetobacter baumannii*	9 (12)	2 (5)
*Enterococcus faecalis/faecium*	4 (5)	0
*Escherichia coli*	8 (11)	0
*Group B Streptococcus*	1 (1)	0
*Haemophilus influenzae*	1 (1)	2 (5)
*Klebsiella pneumoniae*	9 (12)	1 (2)
*Mycobacterium tuberculosis*	0	0
*Pseudomonas aeruginosa*	3 (4)	0
*Staphylococcus aureus*	4 (5)	0
*Streptococcus sangius*	3 (4)	1 (2)
*Streptococcus pneumoniae*	1 (1)	2 (5)
*Ureaplasma urealyticum*	2 (3)	0
Virus		
Cytomegalovirus	5 (7)	3 (7)
Epstein-Barr virus	7 (9)	8 (19)
Herpes-simplex virus	2 (3)	1 (2)
Human herpesvirus 6	2 (3)	0
Fungi		
*Pneumocysttis jirovecii*	0	4 (10)
Mycobacterium tuberculosis (Tested by GeneXpert test)	2/67 (3)	5/39 (13)

Results are n (%).

The post-mortem lung culture results in S5 Table are qualitatively similar, with the frequency of *Escherichia coli* (13% vs 2%) and *Klebsiella pneumoniae* (12% vs 5%) detection being higher in the COVID+ than the COVID- group. Decedents that stayed in hospital for >3 days were more likely to be positive for at least one bacterium compared with hospitalization ≤3 days (58% vs 31% among COVID+ and 52% vs 33% in the COVID- group).

Blood ante-mortem and post-mortem culture results are shown in S6 and S7 Tables, respectively; with *Klebsiella pneumoniae* (11% vs 2%) being more frequently detected post-mortem in COVID+ than in COVID- decedents. Similarly, to lung cultures, the rate of bacterial detection by post-mortem blood culture was higher in those decedents that stayed in the hospital for >3 days compared with ≤3 days (49% vs 22% in COVID+ and 62% vs 10% in COVID-).

Intravascular fibrin-platelet thrombi were found in seven (9%) COVID+ decedents, involving the portal vein in five, hepatic arterioles in one and the hepatic vein also in one (Table 5 and S2 Fig). There was no evidence of definitive sinusoidal microthrombi in the liver. Only one COVID- decedent had fibrin-platelet thrombus in the portal vein. The prevalence of other histopathological changes in the liver was similar in the COVID+ and COVID- decedents, with only cholestasis being more common in COVID+ group (30% vs 7%). A high percentage of COVID+ and COVID- decedents had inflammatory changes in the liver including portal (45% and 54%) and sinusoidal inflammation (54% and 49%). Necrotising granulomatous inflammation overall was found in eight patients (3% COVID+ and 15% of COVID-), five of whom were positive on MTB GeneXpert test (Table 5).

**Table 5:** Histopathology liver features in decedents hospitalized with respiratory illness with (COVID+) and without (COVID‒) SARS-CoV-2 infection.

	COVID+	COVID‒
	N = 74	N = 41
Necrotizing granulomata	2 (3)	6 (15)
Portal inflammation	33 (45)	22 (54)
Interface hepatitis	5 (7)	4 (10)
Sinusoidal inflammation	40 (54)	20 (49)
Lobular hepatitis	10 (14)	5 (12)
Pigment in portal macrophages	1 (1)	1 (2)
Pigment in Kupffer cells	1 (1)	0 (0)
Lympho/erythrophagocytosis in Kupffer cells	9 (12)	3 (7)
Steatosis	24 (32)	9 (22)
Spotty necrosis	11 (15)	2 (5)
Confluent necrosis	4 (5)	2 (5)
Fibrosis	6 (8)	1 (2)
Cholestasis	22 (30)	3 (7)
Congestion	27 (36)	14 (34)
Fibrin-platelet thrombi	7 (9)	1 (2)

Results are n (%).

Table 6 and S3 Fig describes the histological results for the heart. Myocardial fibre hypertrophy (60% and 65%) and interstitial fibrosis (51% and 49%) were the two most common findings in both COVID+ and COVID- groups. Histiocytic infiltrate (9% COVID+ vs 27% COVID-) and myocardial fibre necrosis (10% COVID+ vs 22% COVID-) were described more often in the COVID- than the COVID+ group.

**Table 6:** Histopathology heart features in decedents hospitalized with respiratory illness with (COVID+) and without (COVID‒) SARS-CoV-2 infection.

	COVID+	COVID‒
	n = 68	n = 37
Neutrophilic infiltrate	1 (1)	1 (3)
Lymphocytic infiltrate	6 (9)	6 (16)
Histiocytic infiltrate	6 (9)	10 (27)
Eosinophilc infiltrate	0 (0)	0 (0)
Myocardial fibre necrosis	7 (10)	8 (22)
Vasculitis	0 (0)	0 (0)
Fibrinplatelet thrombi/microthrombi	2 (3)	1 (3)
Interstitial oedema	4 (6)	4 (11)
Myocardial fibre hypertrophy	41 (60)	24 (65)
Interstitial fibrosis	35 (51)	18 (49)

Results are n (%).

## Discussion

This study documents the lung, liver and heart pathologies of decedents with respiratory symptoms at a large hospital in Soweto, South Africa. The findings presented here highlight the severity of the tissue response in the lungs in those patients who are COVID+, including florid Type-II pneumocyte proliferation, with exaggerated cytomegaly and nucleomegaly, and syncytia formation. Exuberant hyaline membrane formation suggests that surfactant production or dysfunction in Type-II pneumocytes may play an important role in the pathogenesis, although there is no current evidence for this [32]. The destructive nature of the inflammatory response resulting in alveolar necrosis and organising pneumonia terminating in increased fibrosis, is emphasised. In these data all measures of Type-II pneumocyte activation were higher among COVID+ than COVID- decedents. Hyaline membrane formation was 50% higher in the COVID+ than COVID- group. These changes reflect the sequence of events that are triggered by SARS-CoV-2 infection of Type-II pneumocytes and possibly Type I pneumocytes, leading to cellular injury, altered surfactant production/function and with coexistent endothelial injury, resulting in capillary leakage, fluid and protein accumulation in alveolar spaces and hyaline membrane formation [22, 33]. Repair follows with Type-II pneumocytes acting as progenitor cells, initially differentiating into transitional Type-II pneumocytes and then into Type-I pneumocytes restoring the alveolar epithelium. Using two different classification methods, the COVD+ decedents displayed substantially higher rates for each stage of damage and repair within the lung. Given the paucity of data from Africa, these results may be used in efforts to inform treatment of severe COVID-19. Clinical trials of surfactant use in ARDS in SARS-CoV-2 infections are currently underway.

Despite important differences in the underlying populations and the methods utilized, this MITS-based study of decedents presenting with symptoms of respiratory disease shows substantial conformity with prior results from other parts of the world concerning lung pathology among persons testing positive for SARS-CoV-2 infection [23, 34].

Comparison of the traditional approach to the classification (Corrin) of the pathological changes seen in acute respiratory distress syndrome with that proposed by Polak et al, highlights a group of decedents, up to 32% of the COVID+ group, that have co-existent vascular pathology in the form of thromboembolic disease [29, 30]. The greater frequency of fibrin platelet thrombi in the COVID+ decedents when compared to COVID- decedents suggests that this is one of the manifestations of this infection and that it is systemic in nature, being found in the pulmonary vasculature, portal vein, hepatic vein and hepatic arterioles. Similar systemic fibrin platelet thrombi have also been found in other series of both adult and paediatric decedents [34, 35]. On the other hand, the Polak et al classification, by not including the exudative phase, misses those COVID+ deaths in the early phase of illness that have only intra-alveolar exudation of fluid, protein and hyaline membrane formation. As would be expected, figures for the remaining groups are similar; regenerative phase (71%), epithelial phase (75%), and repair and fibrotic phases both 68%. COVID- decedents had less histopathological evidence of ARDS in both the traditional and Polak et al classification, suggesting that SARS-CoV-2 infection is a more potent initiator acute respiratory distress syndrome in our population.

The identification of pre-existing small foci of fibrosis in the alveolar septal walls is unexplained but as Soweto is a densely populated urban environment this could be consequent to environmental pollutants including exposure to tobacco smoke or previous episodes of unknown lung disease resulting in early interstitial lung disease [36].

The identification of fibrin-platelet thrombi in the liver suggests that this is a manifestation of a systemic coagulopathy in COVID+ decedents and has also been described by other authors [37]. Although most of the liver findings were similar in the COVID+ and COVID- groups, cholestasis was more common in COVID+ decedents, which might reflect increased levels of hepatic dysfunction in this group [38]. Conversely, necrotising granulomatous inflammation was found more often in the COVID- group, most likely indicative of underlying tuberculosis and reflecting a similar distribution between the two groups as seen in the lungs.

Whilst the findings in the cardiac biopsies show no clear histopathological changes that could be directly attributed to SARS-CoV-2 infection, the changes reflected show evidence of pre-existing chronic cardiac disease which, as a co-morbidity, may impact on cardiac function and final outcome [39].

Three important bacteria, i.e. *Acinetobacter baumannii*, *Escherichia coli* and *Klebsiella pneumoniae* were more often detected in COVID+ decedents, suggesting a potential viral-bacterial association that could have precipitated the death. Moreover, a longer duration of hospitalization before death led to a higher rate of bacterial culture positivity maybe due to nosocomial infections.

Although the number of COVID-19 reported deaths in Africa is low relative to those reported elsewhere, it may be the case that this is an artifact of limitations related to surveillance rather than a lower underlying rate of fatalities in Africa. Other possible reasons could include differences in age-group demographics, the prevalence of underlying comorbidities and factors that could influence the virus inoculum load. Despite these possible differences, the data presented here suggest that the pathological evidence is similar to what has been seen in other parts of the world.

Two aspects of the characteristics shown in Table 1 are of particular interest. The primary noteworthy element is the difference in HIV infection rate between the COVID+ (27%) and COVID- (64%) groups, with an HIV prevalence of 19% among the adult population in the province where the study has been conducted, suggests a similar rate for HIV infection among the COVID+ decedents and the general population [40]. No previous reports were found that provided comparable rates among persons displaying respiratory symptoms. It is unclear to what extent study-specific factors (e.g., whether HIV status was correlated with the likelihood of obtaining consent) are responsible for this result. A second point is the lower rate of MTB among the COVID+ group; this suggests that MTB is not a major co-factor leading to COVID-related deaths in a setting with a high incidence of MTB [41].

Limitations of our study include it describing observed differences among decedents presenting with respiratory disease symptoms based on positive/negative test results for SARS-CoV-2 infection. Entry into the study depended not only on meeting the inclusion criteria, but critically on obtaining informed consent from the relatives of the deceased. It is certainly plausible that decedents for whom consent was obtained differ in important ways from those for whom consent was not obtained. Nevertheless, the ability to gather these data further suggests that the use of the MITS procedure is feasible, and indeed could be used more widely in areas where full autopsies are not widely available or are not culturally appropriate. Also, this was a hypothesis generating descriptive study and no formal statistical analysis was done due to the results’ cell sizes being small. The criteria used to define respiratory symptoms are broad, allowing for the possibility of substantial functionally unobserved heterogeneity among the decedents included in the analyses. Owing to the limited sample size, stratification into more homogeneous groups was not a practical option. Also, the fact that MTB could not be detected by molecular assay detection, but only on GeneXpert test, may suggest that other pathogens also failed detection one the molecular array assay.

Nonetheless, our results suggest that MTB and HIV infections are not associated with fatal COVID-19 pathology. Our study corroborates the feasibility of MITS in adults, and shows that pathogens could be detected with molecular assays, as well as, with culture techniques. Although most indicators of respiratory distress syndrome were undifferentiated between COVID+ and COVID- except for Type-II pneumocytes, more data is needed to support these suggestions.

## Supporting information

S1 Fig Haematoxylin and eosin stained section of lung tissue. Haematoxylin and eosin stained section of lung tissue showing: 1) typical microscopic features of severe SARS-CoV-2 infection, including florid Type-II pneumocyte proliferation in the alveolar spaces, lymphocytic interstitial pneumonitis and extensive necrosis of alveolar septal walls; 2) severe hyaline membrane formation lining the alveolar septa with accompanying interstitial lymphocytic inflammation and Type-II pneumocyte proliferation; 3) cytomegaly, nucleomegaly and multinucleation of Type-II pneumocytes in a background of exuberant intra-alveolar Type-II proliferation (arrow and inset), accompanying intra-alveolar inflammation and early collapse of alveolar septa is also seen; 4) syncytial metaplasia of Type-II pneumocytes; 5) progressive SARS-2-CoV-2 pulmonary injury with hyaline membranes (A), Type-II pneumocyte proliferation (B) and early intra-alveolar organising pneumonia with alveolar septal necrosis (C). A lymphocytic infiltrate is present throughout; 6) multifocal intra-alveolar organising pneumonia; 7) multifocal fibrosis of alveolar septa; 8) “honeycombing” with collagenous thickening of alveolar septa and obliteration of the functional air-blood interface, residual proliferating Type-II pneumocytes remain highlighting the continuum between the different pathological phases of the tissue response in acute respiratory distress syndrome; 9) partial occlusion of a pulmonary vein by fibrin platelet strands (long arrow) contrasting with an area of patency (short arrow); 10) occlusion of pulmonary veins by fibrin platelet thrombi (*) contrasting with adjacent patent pulmonary arterioles.
(PDF)

S2 Fig Haematoxylin and eosin stained section of liver tissue. Haematoxylin and eosin stained section of liver showing intraluminal thrombus in a portal vein radicle (arrow).
(PDF)

S3 Fig Haematoxylin and eosin stained section of heart tissue. Haematoxylin and eosin stained section of heart tissue showing: 1) myocarditis with interstitial lympho-histiocytic inflammatory infiltrate, interstitial oedema and focal myocardial fibre disruption; 2) lympho-histiocytic myocarditis (short arrow) and myocardial fibre disruption and necrosis (long arrows); 3) focus of myocardial fibre necrosis (arrow) accompanied by lympho-histiocytic inflammatory infiltration; 4) intra-capillary fibrin-platelet thrombi in interstitial vasculature (arrows).
(PDF)

S1 Table List of candidate pathogens used with the respiratory open array system. (DOCX)

S2 Table Histopathology lung features in SARS-CoV-2 infected decedents stratified by HIV infection status. (DOCX)

S3 Table Histopathology lung features in SARS-CoV-2 infected decedents stratified by length of hospital stay. (DOCX)

S4 Table Organisms identified on post-mortem lung molecular assay detection in SARS-CoV-2 infected decedents stratified by HIV infection status. (DOCX)

S5 Table Bacteria identified on post-mortem lung culture in decedents hospitalized with respiratory illness with (COVID+) and without (COVID-) SARS-CoV-2 infection. (DOCX)

S6 Table Bacteria identified on ante-mortem blood culture. (DOCX)

S7 Table Bacteria identified on post-mortem blood culture. (DOCX)
