# Supplementary material for: Clinical characteristics and histopathology of COVID-19 related deaths in South African adults
Source: PLoS One. 2022 Jan 20;17(1):e0262179. doi: 10.1371/journal.pone.0262179 (PMC8775212; doi:10.1371/journal.pone.0262179)
Supplement: S1 Fig — Haematoxylin and eosin stained section of lung tissue showing: 1) typical microscopic features of severe SARS-CoV-2 infection, including florid Type-II pneumocyte proliferation in the alveolar spaces, lymphocytic interstitial pneumonitis and extensive necrosis of alveolar septal walls; 2) severe hyaline membrane formation lining the alveolar septa with accompanying interstitial lymphocytic inflammation and Type-II pneumocyte proliferation; 3) cytomegaly, nucleomegaly and multinucleation of Type-II pneumocytes in a background of exuberant intra-alveolar Type-II proliferation (arrow and inset), accompanying intra-alveolar inflammation and early collapse of alveolar septa is also seen; 4) syncytial metaplasia of Type-II pneumocytes; 5) progressive SARS-2-CoV-2 pulmonary injury with hyaline membranes (A), Type-II pneumocyte proliferation (B) and early intra-alveolar organising pneumonia with alveolar septal necrosis (C). A lymphocytic infiltrate is present throughout; 6) multifocal intra-alveolar organising pneumonia; 7) multifocal fibrosis of alveolar septa; 8) “honeycombing” with collagenous thickening of alveolar septa and obliteration of the functional air-blood interface, residual proliferating Type-II pneumocytes remain highlighting the continuum between the different pathological phases of the tissue response in acute respiratory distress syndrome; 9) partial occlusion of a pulmonary vein by fibrin platelet strands (long arrow) contrasting with an area of patency (short arrow); 10) occlusion of pulmonary veins by fibrin platelet thrombi (*) contrasting with adjacent patent pulmonary arterioles. (PDF) [file pone.0262179.s001.pdf]

S1 Fig: Haematoxylin and eosin stained section of lung tissue

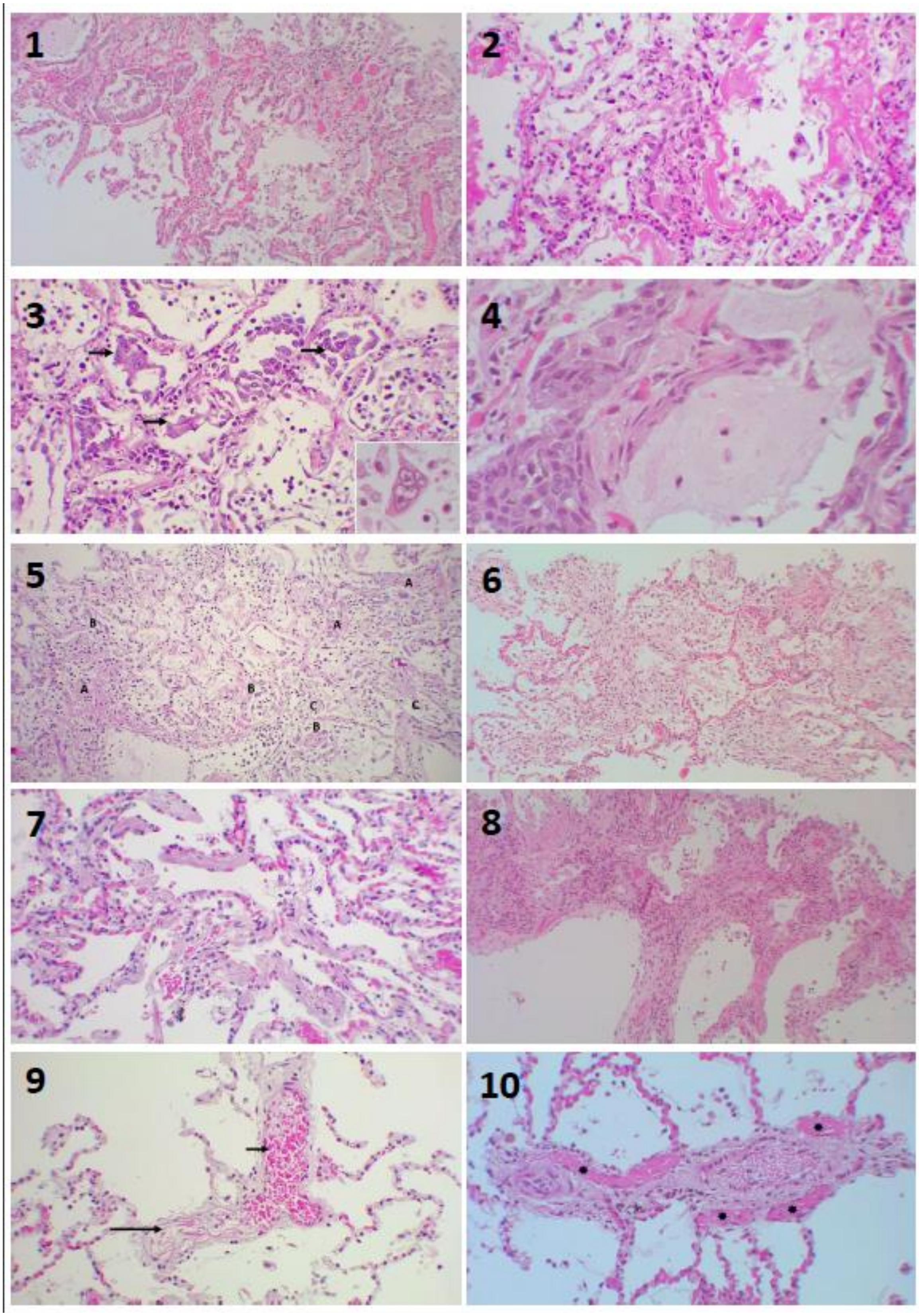

Haematoxylin and eosin stained section of lung tissue showing: 1) typical microscopic features of severe SARS-CoV-2 infection, including florid Type-II pneumocyte proliferation in the alveolar spaces, lymphocytic interstitial pneumonitis and extensive necrosis of alveolar septal walls; 2) severe hyaline membrane formation lining the alveolar septa with accompanying interstitial lymphocytic inflammation and Type-II pneumocyte proliferation; 3) cytomegaly, nucleomegaly and multinucleation of Type-II pneumocytes in a background of exuberant intra-alveolar Type-II proliferation (arrow and inset), accompanying intra-alveolar inflammation and early collapse of alveolar septa is also seen; 4) syncytial metaplasia of Type-II pneumocytes; 5) progressive SARS-2-CoV-2 pulmonary injury with hyaline membranes (A), Type-II pneumocyte proliferation (B) and early intra-alveolar organising pneumonia with alveolar septal necrosis (C). A lymphocytic infiltrate is present throughout; 6) multifocal intra-alveolar organising pneumonia; 7) multifocal fibrosis of alveolar septa; 8) “honeycombing” with collagenous thickening of alveolar septa and obliteration of the functional air-blood interface, residual proliferating Type-II pneumocytes remain highlighting the continuum between the different pathological phases of the tissue response in acute respiratory distress syndrome; 9) partial occlusion of a pulmonary vein by fibrin platelet strands (long arrow) contrasting with an area of patency (short arrow); 10) occlusion of pulmonary veins by fibrin platelet thrombi (*) contrasting with adjacent patent pulmonary arterioles.
