# Supplementary material for: Clinical characteristics and histopathology of COVID-19 related deaths in South African adults
Source: PLoS One. 2022 Jan 20;17(1):e0262179. doi: 10.1371/journal.pone.0262179 (PMC8775212; doi:10.1371/journal.pone.0262179)
Supplement: S2 Fig — Haematoxylin and eosin stained section of liver showing intraluminal thrombus in a portal vein radicle (arrow). (PDF) [file pone.0262179.s002.pdf]

S2 Fig: Haematoxylin and eosin stained section of liver tissue

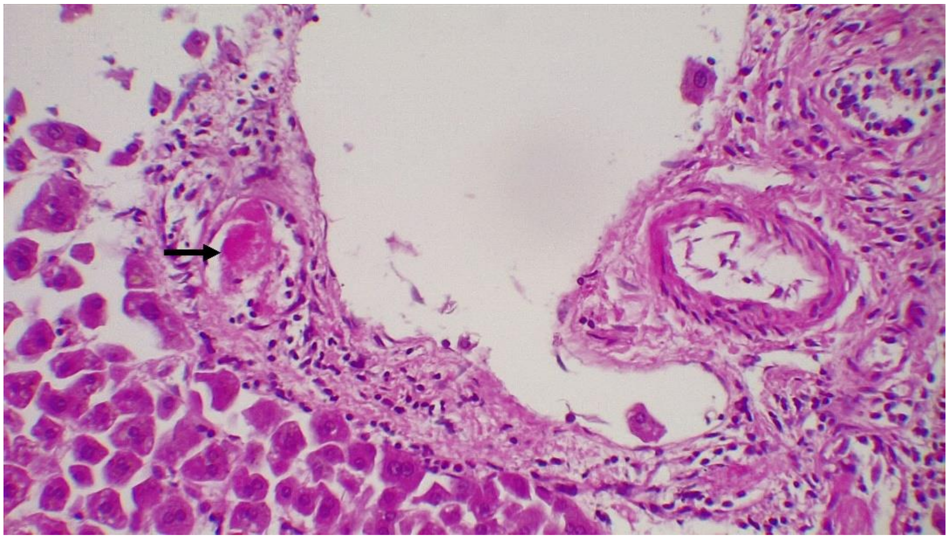

Haematoxylin and eosin stained section of liver showing intraluminal thrombus in a portal vein radicle (arrow).
