# Supplementary material for: Clinical characteristics and histopathology of COVID-19 related deaths in South African adults
Source: PLoS One. 2022 Jan 20;17(1):e0262179. doi: 10.1371/journal.pone.0262179 (PMC8775212; doi:10.1371/journal.pone.0262179)
Supplement: S3 Fig — Haematoxylin and eosin stained section of heart tissue showing: 1) myocarditis with interstitial lympho-histiocytic inflammatory infiltrate, interstitial oedema and focal myocardial fibre disruption; 2) lympho-histiocytic myocarditis (short arrow) and myocardial fibre disruption and necrosis (long arrows); 3) focus of myocardial fibre necrosis (arrow) accompanied by lympho-histiocytic inflammatory infiltration; 4) intra-capillary fibrin-platelet thrombi in interstitial vasculature (arrows). (PDF) [file pone.0262179.s003.pdf]

S3 Fig: Haematoxylin and eosin stained section of heart tissue

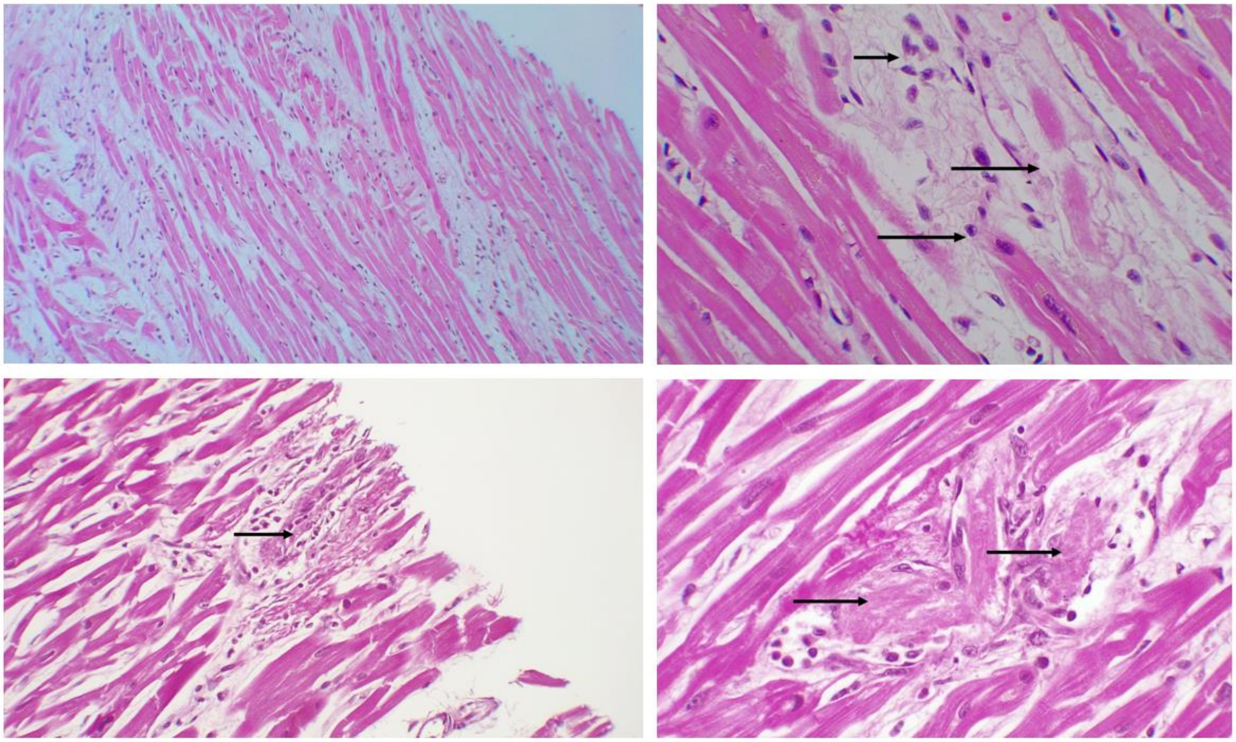

Haematoxylin and eosin stained section of heart tissue showing: 1) myocarditis with interstitial lympho-histiocytic inflammatory infiltrate, interstitial oedema and focal myocardial fibre disruption; 2) lympho-histiocytic myocarditis (short arrow) and myocardial fibre disruption and necrosis (long arrows); 3) focus of myocardial fibre necrosis (arrow) accompanied by lympho-histiocytic inflammatory infiltration; 4) intra-capillary fibrin-platelet thrombi in interstitial vasculature (arrows).
