# Supplementary material for: Clinical characteristics and histopathology of COVID-19 related deaths in South African adults
Source: PLoS One. 2022 Jan 20;17(1):e0262179. doi: 10.1371/journal.pone.0262179 (PMC8775212; doi:10.1371/journal.pone.0262179)
Supplement: S1 Table — (DOCX) [file pone.0262179.s004.docx]

**S1 Table: List of candidate pathogens used with the Respiratory Open Array system**

| *Acinetobacter baumanii* |
| --- |
| Adenovirus |
| Bocavirus |
| *Bordetella pertussis* |
| *Bordetella spp.* |
| *Chlamydia trachomatis* |
| *Chlamydophila pneumoniae* |
| Candida albicans |
| Candida parapsilosis |
| *Coxiella burnetii* |
| Cytomegalovirus |
| Entereovirus_D68 |
| Entereovirus_pan |
| Epstein-Barr virus |
| *Enterococcus faecalis/faecium* |
| *Escherichia coli* |
| *Group A Streptococcus-Streptococcus pyogenes* |
| *Group B Streptococcus* |
| *Haemophilus influenzae* |
| *H. influenzae* Type B |
| Herpes-simplex virus -1 |
| Herpes-simplex virus -2 |
| Human Herpesvirus 6 |
| Human metapneumovirus |
| Influenza A |
| Influenza B |
| Influenza C |
| *Klebsiella pneumoniae* |
| *Legionella pneumophila* |
| *Listeria monocytogenes* |
| Measles |
| *Moraxella catarrhalis* |
| *Mumps* |
| *Mycobacterium tuberculosis* |
| *Neisseria meningitidis* |
| Parainfluenza virus type 1 |
| Parainfluenza virus type 2 |
| Parainfluenza virus type 3 |
| *Parechovirus* |
| *Pneumocystis jirovecii* |
| *Pseudomonas aeruginosa* |
| Respiratory syncytical virus (RSV) – A |
| Respiratory syncytical virus (RSV) – B |
| Rhinovirus |
| Rubella |
| Salmonella enterica |
| *Staphylococcus aureus* |
| *Streptococcus sanguis* |
| *Streptococcus pneumoniae* |
| *Ureaplasma urealyticum* |
| Varicella virus |
