# Supplementary material for: Clinical characteristics and histopathology of COVID-19 related deaths in South African adults
Source: PLoS One. 2022 Jan 20;17(1):e0262179. doi: 10.1371/journal.pone.0262179 (PMC8775212; doi:10.1371/journal.pone.0262179)
Supplement: S2 Table — (DOCX) [file pone.0262179.s005.docx]

**S2 Table: Histopathology lung features in SARS-CoV-2 infected decedents stratified by HIV infection status**

|  | COVID positive | |
| --- | --- | --- |
|  | HIV-infected | HIV-uninfected |
|  | n = 20 | n = 55 |
| Necrotizing granulomata | 3 (15) | 2 (4) |
| Aspirated material | 1 (5) | 0 (0) |
| Neutrophilic infiltrate | 3 (15) | 17 (31) |
| Intra-alveolar hemosiderosis | 2 (10) | 10 (18) |
| Congestion of alveolar septa | 16 (80) | 50 (91) |
| Alveolar septal oedema | 17 (85) | 43 (78) |
| Interstitial inflammation | 19 (95) | 48 (87) |
| Intravascular fibrin/microthrombi | 6 (30) | 19 (35) |
| Megakaryocytes | 13 (65) | 30 (55) |
| Intra-alveolar hemorrhage | 4 (20) | 11 (20) |
| Intra-alveolar oedema | 3 (15) | 13 (24) |
| Intra-alveolar fibrin | 12 (60) | 21 (38) |
| Hyaline membranes | 11 (55) | 35 (64) |
| Alveolar collapse | 10 (50) | 38 (69) |
| Type II pneumocyte overall | 16 (80) | 45 (82) |
| Increased alveolar macrophages | 16 (80) | 47 (85) |
| Alveolar septal necrosis | 15 (75) | 46 (84) |
| Intra-alveolar or Septal collagen or honeycombing | 18 (90) | 42 (76) |

Results are n (%).
