# Supplementary material for: Clinical characteristics and histopathology of COVID-19 related deaths in South African adults
Source: PLoS One. 2022 Jan 20;17(1):e0262179. doi: 10.1371/journal.pone.0262179 (PMC8775212; doi:10.1371/journal.pone.0262179)
Supplement: S3 Table — (DOCX) [file pone.0262179.s006.docx]

**S3 Table: Histopathology lung features in SARS-CoV-2 infected decedents stratified by length of hospital stay**

|  | COVID positive | |
| --- | --- | --- |
|  | Hospital stay less or equal to 3 days | Hospital stay greater than 3 days |
|  | n = 32 | n = 43 |
| Necrotizing granulomata | 1 (3) | 4 (9) |
| Aspirated material | 0 (0) | 1 (2) |
| Neutrophilic infiltrate | 5 (16) | 15 (35) |
| Intra-alveolar hemosiderosis | 0 (0) | 12 (28) |
| Congestion of alveolar septa | 28 (88) | 38 (88) |
| Alveolar septal oedema | 24 (75) | 36 (84) |
| Interstitial inflammation | 28 (88) | 39 (91) |
| Intravascular fibrin/microthrombi | 11 (34) | 14 (33) |
| Megakaryocytes | 21 (66) | 22 (51) |
| Intra-alveolar hemorrhage | 6 (19) | 9 (21) |
| Intra-alveolar oedema | 6 (19) | 10 (23) |
| Intra-alveolar fibrin | 15 (47) | 18 (42) |
| Hyaline membranes | 22 (69) | 24 (56) |
| Alveolar collapse | 20 (63) | 28 (65) |
| Combined Type II | 25 (78) | 36 (84) |
| Increased alveolar macrophages | 24 (75) | 39 (91) |
| Alveolar septal necrosis | 24 (75) | 37 (86) |
| Intra-alveolar or Septal collagen or honeycombing | 24 (75) | 36 (84) |

Results are n (%).
