# Supplementary material for: Clinical characteristics and histopathology of COVID-19 related deaths in South African adults
Source: PLoS One. 2022 Jan 20;17(1):e0262179. doi: 10.1371/journal.pone.0262179 (PMC8775212; doi:10.1371/journal.pone.0262179)
Supplement: S4 Table — (DOCX) [file pone.0262179.s007.docx]

**S4 Table:** **Organisms identified on post-mortem lung molecular assay detection in SARS-CoV-2 infected decedents stratified by HIV infection status**

|  | COVID positive | |
| --- | --- | --- |
|  | HIV-infected  N=20 | HIV-uninfected  N=55 |
| Bacteria |  |  |
| *Acinetobacter baumannii* | 3 (15) | 6 (11) |
| *Enterococcus faecalis/faecium* | 1 (5) | 3 (5) |
| *Escherichia coli* | 3 (15) | 5 (9) |
| *Streptococcus agalactiae* | 1 (5) | 0 (0) |
| *Haemophilus influenzae* | 0 (0) | 1 (2) |
| *Klebsiella pneumoniae* | 2 (10) | 7 (13) |
| *Pseudomonas aeruginosa* | 2 (10) | 1 (2) |
| *Staphylococcus aureus* | 1 (5) | 3 (5) |
| *Streptococcus sangius* | 0 (0) | 3 (5) |
| *Streptococcus pneumoniae* | 1 (5) | 0 (0) |
| *Ureaplasma urealyticum* | 1 (5) | 1 (2) |
| Virus |  |  |
| Cytomegalovirus | 3 (15) | 2 (4) |
| Epstein-Barr virus | 3 (15) | 4 (7) |
| Herpes-simplex virus | 1 (5) | 1 (2) |
| Human herpesvirus 6 | 0 (0) | 2 (4) |
| Mycobacterium tuberculosis (Tested by GenXpert test) | 2/18 (11) | 0/49 (0) |

Results are n (%).
