# Supplementary material for: Clinical characteristics and histopathology of COVID-19 related deaths in South African adults
Source: PLoS One. 2022 Jan 20;17(1):e0262179. doi: 10.1371/journal.pone.0262179 (PMC8775212; doi:10.1371/journal.pone.0262179)
Supplement: S6 Table — (DOCX) [file pone.0262179.s009.docx]

**S6 Table: Bacteria identified on ante-mortem blood culture**

|  |  | COVID+ |  |  | COVID- |  |
| --- | --- | --- | --- | --- | --- | --- |
|  | Overall | Hospital stay less or equal to 3 days | Hospital stay greater than 3 days | Overall | Hospital stay less or equal to 3 days | Hospital stay greater than 3 days |
|  | n = 60 | n = 27 | n = 33 | n = 29 | n = 11 | n = 18 |
| *Acinetobacter baumannii* | 1 (2) | 0 (0) | 1 (3) | 2 (7) | 1 (9) | 1 (6) |
| *Enterococcus faecalis* | 2 (3) | 0 (0) | 2 (6) | 0 (0) | 0 (0) | 0 (0) |
| *Enterococcus faecium* | 1 (2) | 0 (0) | 1 (3) | 0 (0) | 0 (0) | 0 (0) |
| *Escherichia coli* | 1 (2) | 0 (0) | 1 (3) | 0 (0) | 0 (0) | 0 (0) |
| *Klebsiella pneumoniae* | 2 (3) | 1 (4) | 1 (3) | 0 (0) | 0 (0) | 0 (0) |
| *Proteus vulgaris* | 1 (2) | 1 (4) | 0 (0) | 0 (0) | 0 (0) | 0 (0) |
| *Pseudomonas aeruginosa* | 3 (5) | 1 (4) | 2 (6) | 0 (0) | 0 (0) | 0 (0) |
| *Staphylococcus aureus* | 0 (0) | 0 (0) | 0 (0) | 1 (3) | 0 (0) | 1 (6) |

Results are n (%).

Furthermore, Corynebacterium species, Micrococcus species*,* Coagulase negative staphylococcus, *Corynebacterium spp* and *Cryptococcus neoformans*, were identified but were considered contaminants and to be likely of non-clinical significance.
