# Supplementary material for: Clinical characteristics and histopathology of COVID-19 related deaths in South African adults
Source: PLoS One. 2022 Jan 20;17(1):e0262179. doi: 10.1371/journal.pone.0262179 (PMC8775212; doi:10.1371/journal.pone.0262179)
Supplement: S7 Table — (DOCX) [file pone.0262179.s010.docx]

**S7 Table: Bacteria identified on post-mortem blood culture**

|  | COVID+ |  |  | COVID- |  |  |
| --- | --- | --- | --- | --- | --- | --- |
|  | Overall | Hospital stay less or equal to 3 days | Hospital stay greater than 3 days | Overall | Hospital stay less or equal to 3 days | Hospital stay greater than 3 days |
|  | n = 75 | n = 32 | n = 43 | n = 42 | n = 21 | n = 21 |
| Positive for at least one bacterium likely to be pathogen | 28 (37.3) | 7 (22) | 21 (49) | 15 (35.7) | 2 (10) | 13 (62) |
| *Acinetobacter baumannii* | 4 (5.3) | 0 (0) | 4 (9) | 3 (7.1) | 0 (0) | 3 (14) |
| *Candida albicans* | 5 (6.7) | 2 (6) | 3 (7) | 2 (4.8) | 2 (10) | 0 (0) |
| *Candida glabrata* | 2 (2.6) | 0 (0) | 2 (5) | 0 (0) | 0 (0) | 0 (0) |
| *Enterococcus faecalis* | 3 (4) | 1 (3) | 2 (5) | 2 (4.8) | 0 (0) | 2 (10) |
| *Enterococcus faecium* | 4 (5.3) | 0 (0) | 4 (9) | 5 (11.9) | 0 (0) | 5 (24) |
| *Escherichia coli* | 7 (9.3) | 3 (9) | 4 (9) | 3 (7.1) | 0 (0) | 3 (14) |
| *Klebsiella oxytoca* | 0 (0) | 0 (0) | 0 (0) | 2 (4.8) | 1 (5) | 1 (5) |
| *Klebsiella pneumoniae* | 8 (10.7) | 1 (3) | 7 (16) | 1 (2.4) | 0 (0) | 1 (5) |
| *Proteus mirabilis* | 4 (5.3) | 0 (0) | 4 (9) | 1 (2.4) | 0 (0) | 1 (5) |
| *Pseudomonas aeruginosa* | 1 (1.3) | 0 (0) | 1 (2) | 0 (0) | 0 (0) | 0 (0) |
| *Staphylococcus aureus* | 3 (4) | 2 (6) | 1 (2) | 1 (2.4) | 0 (0) | 1 (5) |
| *Streptococcus agalactiae* | 1 (1.3) | 1 (3) | 0 (0) | 0 (0) | 0 (0) | 0 (0) |

Results are n (%).

Furthermore, Serratia species, other Bacillus species, *Citrobacter freundii, Citrobacter koseri,* Coagulase Negative Staphylococcus, Corynebacterium spp, *Enterobacter cloacae, Stenotrophomonas maltophilia* and *Streptococcus viridans*, were identified but were considered contaminants and to be likely of non-clinical significance.
